# Accuracy of Detecting Atrial Fibrillation: A Systematic Review and Meta-Analysis of Wrist-Worn Wearable Technology

**DOI:** 10.7759/cureus.20362

**Published:** 2021-12-12

**Authors:** Seema Belani, Waseem Wahood, Patrick Hardigan, Andon N Placzek, Stephen Ely

**Affiliations:** 1 College of Allopathic Medicine, Dr. Kiran C. Patel College of Allopathic Medicine, Davie, USA; 2 Medicine, Nova Southeastern University Dr. Kiran C. Patel College of Allopathic Medicine, Davie, USA; 3 Health Professions Division, Nova Southeastern University Dr. Kiran C. Patel College of Allopathic Medicine, Davie, USA; 4 Medical Education and Simulation, Dr. Kiran C. Patel College of Allopathic Medicine, Fort Lauderdale, USA; 5 Cardiothoracic Surgery, Nova Southeastern University Dr. Kiran C. Patel College of Allopathic Medicine, Fort Lauderdale, USA

**Keywords:** detection, ecg, specificity, sensitivity, accuracy, wearables, atrial fibrillation

## Abstract

Atrial fibrillation (AF) is the most commonly diagnosed arrhythmia, and ECG remains the gold standard for diagnosing AF. Wrist-worn technologies are appealing for their ability to passively process near-continuous pulse signals. The clinical application of wearable devices is controversial. Our systematic review and meta-analysis qualitatively and quantitatively analyze available literature on wrist-worn wearable devices (Apple Watch, Samsung, and KardiaBand) and their sensitivity and specificity in detecting AF compared to conventional methods. Preferred Reporting Items for Systematic Reviews and Meta-Analyses (PRISMA) guidelines were followed, yielding nine studies (n = 1,581). Observational studies assessing the sensitivity and specificity of wrist-worn wearables in detecting AF in patients with and without a history of AF were included and analyzed using a fixed-effect model with an inverse-variance method. In patients with a history of AF, the overall sensitivity between device groups did not significantly differ (96.83%; P = 0.207). Specificity significantly differed between Apple, Samsung, and KardiaBand (99.61%, 81.13%, and 97.98%, respectively; P<0.001). The effect size for this analysis was highest in the Samsung device group. Two studies (n = 796) differentiated cohorts to assess device sensitivity in patients with known AF and device specificity in patients with normal sinus rhythm (NSR) (sensitivity: 96.02%; confidence intervals (CI) 93.85%-97.59% and specificity: 98.82%; CI:97.46%-99.57%). Wrist-worn wearable devices demonstrate promising results in detecting AF in patients with paroxysmal AF. However, more rigorous prospective data is needed to understand the limitations of these devices in regard to varying specificities which may lead to unintended downstream medical testing and costs.

## Introduction and background

Atrial fibrillation (AF) is the most commonly diagnosed arrhythmia in clinical practice [1]. It is estimated that 2.3 million adults in the United States are burdened by AF, and as the population ages that number is expected to increase to 5.6 million by 2050 [1]. The consequences of AF, including thromboembolic events, stroke, and heart failure, are well documented. These consequences of disease progression account for the significant impact on morbidity, mortality, and healthcare costs [1]. Therefore, AF is not only a devastating clinical problem but also a public health and economic burden.

While AF typically presents with palpitations, dyspnea, chest pain, and fatigue, it is estimated that a 10-40% incidence of AF is asymptomatic [2]. Subclinical or unrecognized AF presents with the same risks as symptomatic AF and has critical implications when first manifesting at the time of acute stroke. The relationship between arrhythmia and stroke is perplexing; however, reports from the Framingham Study have demonstrated that the concomitant presentation of stroke with newly diagnosed AF suggests that cardiac emboli may be an important cause of stroke [3-4]. Furthermore, the temporal relationship between AF and stroke highlights the importance of prophylactic measures for stroke prevention [4]. Early detection of both clinical and subclinical AF allows for early preventative measures, which would improve health outcomes.

Interpretation of a 12-lead electrocardiogram (ECG) by a trained cardiologist or heart rhythm specialist is the gold standard for detecting AF [5]. The 2014 guidelines from the American Heart Association/American Stroke Association recommend screening for AF with pulse assessments during routine clinical visits and subsequent 12-lead ECGs among individuals who demonstrate an irregular pulse [6-7]. The guidelines highlight the advantages of active screening in patients >65, however, they lack recommendations on frequency [6-7]. Similarly, the US Preventive Services Task Force (USPSTF) published a statement that the current evidence is insufficient to evaluate the benefit of screening for AF with ECG [8]. The problem is that too many uncertainties exist to warrant routine ECG testing for all patients, especially those who are not high-risk. Current research points to the low prevalence and high costs as significant contributors to such screening challenges [9]. One challenge is that ambulatory ECG monitoring, ranging from 12 hours to 14 days, is only marginally representative of a patient’s experience due to the unpredictable and sporadic nature of AF [10].

While it is clear that more evidence is needed to illustrate the advantages of early screening protocols, studies have demonstrated that active screening for undiagnosed AF has proven to be effective starting at an age of 40 years [11]. Furthermore, screening with ECG can identify patients with asymptomatic AF [12]. Therefore, early detection leads to potentially reducing the risk of stroke and heart failure in patients with AF.

An increasing number of individuals use commercially available wearable technology, which has paralleled innovation in the mobile health (mHealth) space. mHealth has been an avenue for expanding AF detection beyond traditional cardiac telemetry. Currently, mHealth technology utilizes electrocardiographic or photoplethysmographic (PPG) signal processing to detect AF [13]. While ECG remains the gold standard for AF detection, these novel technologies are appealing for their ability to passively process near-continuous pulse signals [13]. PPG and similar technology offer an inexpensive and non-invasive means for continuous monitoring throughout the cardiac cycle. Developing the accuracy of wearable technology has the potential to eliminate some of the challenges observed with conventional screening methods for AF detection.

Objective

Observational clinical studies measuring the accuracy of wearable devices in detecting AF demonstrate promising outcomes. This novel development has garnered significant interest in the field of cardiology over the past five years due to the recent FDA clearance of multiple mobile technologies for AF detection [13]. However, the reported accuracy of wrist-worn wearable technologies is inconsistent across the literature.

To date, there are several reviews that look at the use of wearable devices for the detection of AF [14-15]. Only one systematic review focused on the sensitivity and specificity of wearable devices in detecting AF [15]. Therefore, we conducted a systematic review and meta-analysis to compare the accuracy of the most recent wrist-worn wearable devices in detecting AF. Our objective was to qualitatively and quantitatively analyze the available literature on wrist-worn wearable devices and their sensitivity and specificity in detecting AF compared to conventional methods.

## Review

Methods

This systematic review and meta-analysis was conducted using the PICO (Patient, Intervention, Comparator, and Outcome) method and followed the framework outlined in the Preferred Reporting Items for Systematic Reviews and Meta-Analyses (PRISMA) guidelines [16].

Search strategy

A comprehensive search of several databases from each database’s inception to July 27th, 2020, English language, was conducted. The databases included Ovid MEDLINE(R) and Epub Ahead of Print, In-Process & Other Non-Indexed Citations, and Daily, Ovid EMBASE, Ovid Cochrane Central Register of Controlled Trials, Ovid Cochrane Database of Systematic Reviews, and Scopus. The search strategy was designed and conducted by an experienced librarian with input from two authors (S.B and W.W). Controlled vocabulary supplemented with keywords was used to search for data collection accuracy of wearables and their efficacy in predicting outcomes in AF.

Study selection criteria

Studies were included for review if they met the following criteria: involve human subjects, collect EKG data, assess the accuracy of wrist-worn wearables, diagnose atrial fibrillation, and published within the past five years (2016-2020), due to the increase in digital health technologies since the FDA approval for the AliveCor Kardia device as the first wearable technology to detect AF [17]. Studies were excluded based on the following predefined criteria: non-English language, pediatric population, mobile apps, and data that lacked sensitivity and specificity statistics.

Quality assessment

The quality of outcomes was assessed using the GRADE (Grading of Recommendations, Assessment, Development, and Evaluations) methodology, while studies’ quality of evidence was assessed using the modified Newcastle-Ottawa Scale [18,19].

Data extraction

Eligible studies were pooled according to the aforementioned inclusion and exclusion criteria. Data extraction from articles, tables, and figures was pulled by one reviewer (S.B) and accuracy of data entry was verified by a second reviewer (W.W). Data collected included: study author, year of publication, type of device used, sample size, number of recorded events, method of AF verification, true positive, true negative, false positive, false negative, specificity, and sensitivity (Table 1).

**Table 1:** Summary of Data Extraction and Study Characteristics SR: sinus rhythm, AF: atrial fibrillation, ECG: electrocardiogram, ICM: implantable cardiac monitor, TP: true positive, TN: true negative, FP: false positive, FN: false negative

Study	Year	Device Group	Sample Size (n)	Female (n)	Recorded Events	Past Medical History	Method of AF Verification	TP	TN	FP	FN	Sensitivity	Specificity
Seshadri et al. [20]	2020	Apple	50		284	Undergone cardiac surgery	Telemetry	81	200	0	3	96.40%	100%
Apple, Inc [21]	2018	Apple	588		479	301 AF 287 SR	12-lead ECG	236	238	1	4	98.30%	99%
Tison et al. [22]	2018	Apple	51	8	51	AF	12-lead ECG	40	9	1	1	97.56%	90%
Wasserlauf et al. [23]	2019	KB	24	9	82	Paroxysmal AF	ICM recording	80	N/A	N/A	2	97.56%	N/A
Bumgarner et al. [24]	2018	KB	100	17	169	AF	ECG	63	37	7	5	92.65%	97.57%
Rajakariar et al. [25]	2020	KB	200	43	191 (9/200 no analysis)	38 AF 162 SR	12-lead ECG	47	113	28	3	94%	80.14%
Dorr et al. [26]	2018	Samsung	508	225	508	271 AF 237 SR	Cardiologist interpretation of iECG from kardiamobile	222	266	5	15	93.67%	98.15%
Ding et al. [27]	2019	Samsung	40	8	314	9 AF 30 SR	7-lead Holter monitor	54	254	5	1	98.20%	98.07%
Bashar et al. [28]	2019	Samsung	20		242	8AF 12 SR	7-lead Holter monitor	50	185	5	2	96.15%	97.37%

Statistical analysis

A meta-analysis of diagnostic test specificity and sensitivity was conducted, with results represented as effect sizes (ES) with corresponding 95% confidence intervals (CIs). A fixed-effect model with an inverse-variance method was used [29-31]. Heterogeneity between groups represents the statistical difference between the three groups in their respective outcomes. Funnel plots were created to assess publication bias within studies. Statistical analysis was done using STATA 16.0 (Stata-Corp 2020. STATA Statistical Software: Release 15. College Station, TX: StataCorp LP) and its “metan” and “metafunnel” packages. A P-value <0.05 was considered significant.

Results

Search Results

**Figure 1:**
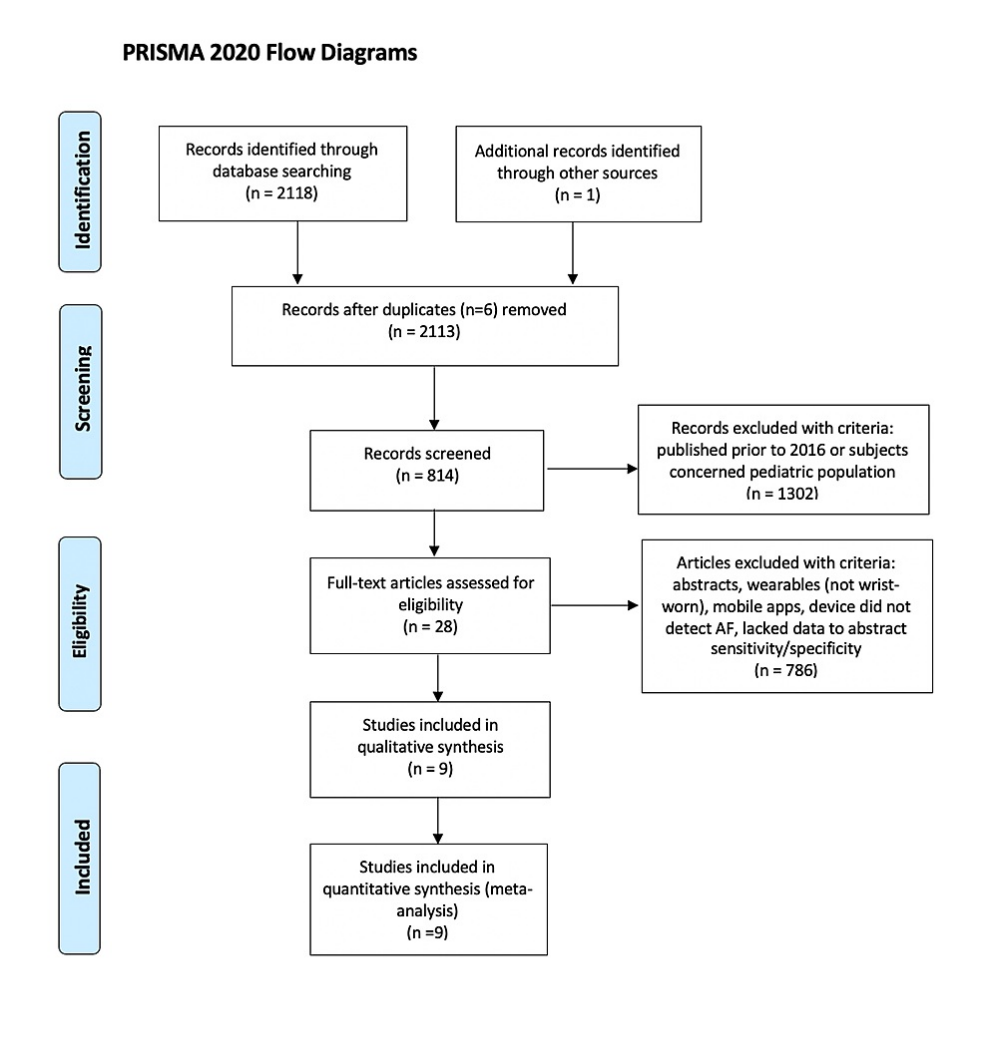
Preferred Reporting Items for Systematic Reviews and Meta-Analyses (PRISMA) search strategy flowchart of the present systematic review and meta-analysis AF: atrial fibrillation

Our search strategy yielded a total of 2113 unique articles. After removal of 1263 articles that were published prior to 2016 and 39 articles that concerned a pediatric population, inclusion/exclusion criteria were applied to abstracts of the remaining 814 articles. This resulted in 28 articles that underwent full-text analysis, of which nine met the predefined eligibility criteria and were included in the qualitative and quantitative synthesis (Figure 1) [20-28].

Characteristics of studies

The total number of studies included was nine, with three regarding Apple devices, three regarding KardiaBand (KB), and three regarding Samsung devices [20-28]. In total, 1629 patients were included in this meta-analysis and an average of 259 instances were recorded per study. Mean age of all studies was 70.2. Percentage of females across all studies was 29.18% (Table 1).

Sensitivity

**Figure 2:**
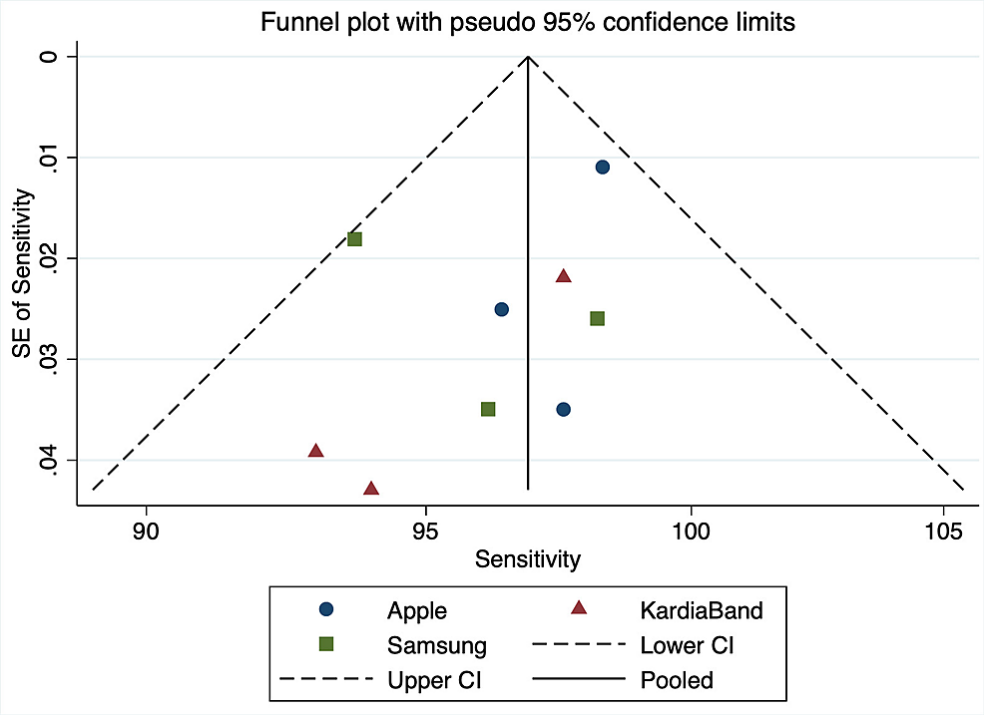
Sensitivity Funnel Plot SE: standard error, CI: confidence interval

**Figure 3:**
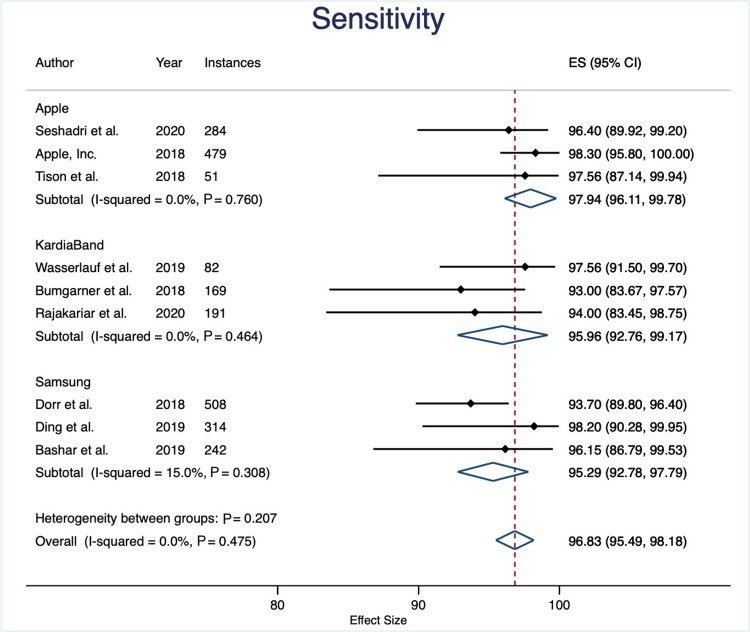
Sensitivity Forest Plot

The overall sensitivity between device groups was not statistically significant (P = 0.276). Apple devices had an average sensitivity of 97.9% (95% CI: 96.1% to 99.7%), KB with 96.9% (95% CI: 94.7% to 99.2%), and Samsung devices with 95.5% (95% CI: 93.1% to 97.8%). Overall sensitivity across all devices was 97.0% (95% CI: 95.8% to 98.2%) (Figures 2-3).

Specificity

**Figure 4:**
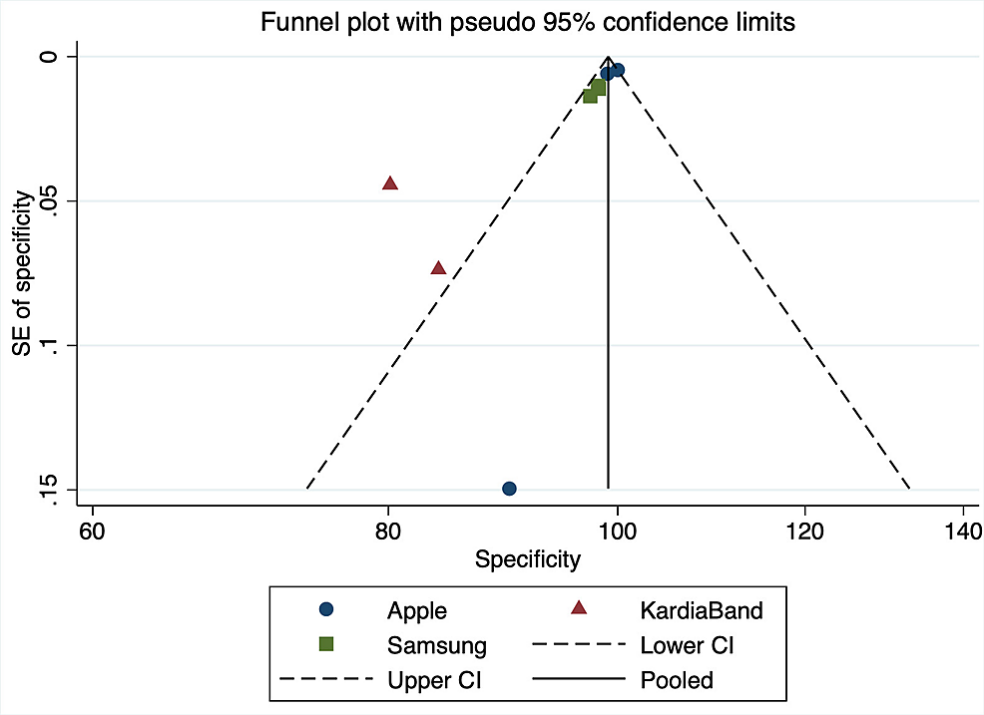
Specificity Funnel Plot SE: standard error, CI: confidence interval

**Figure 5:**
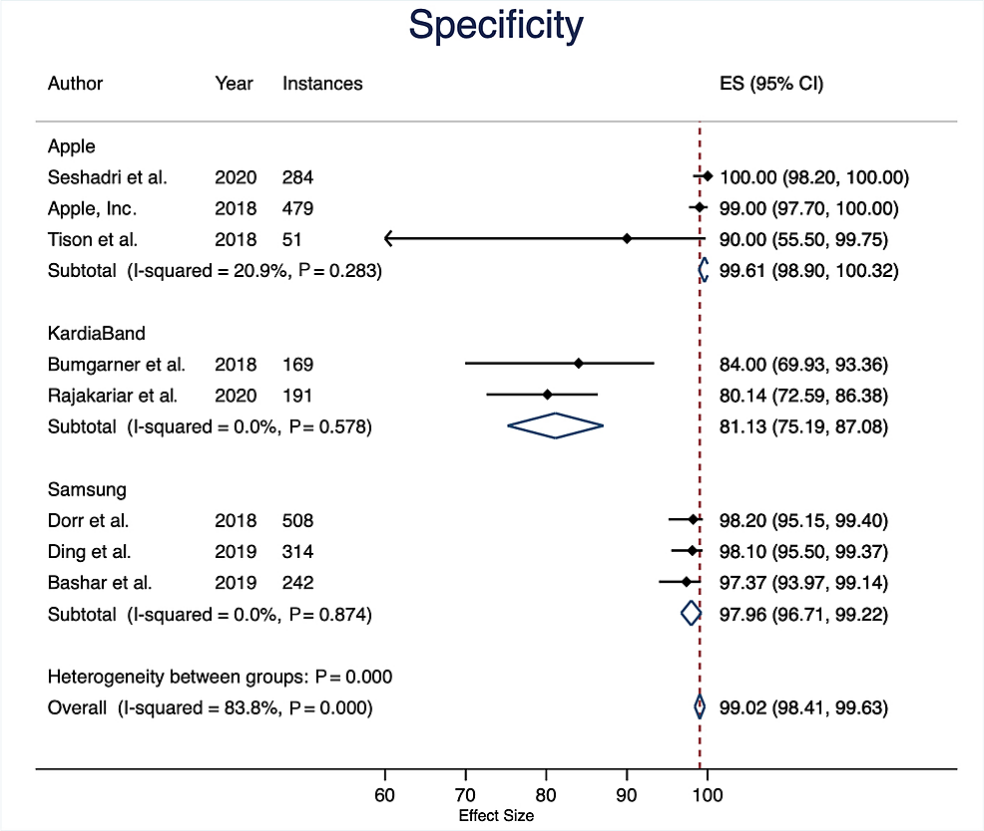
Specificity Forest Plot

The overall specificity between device groups was statistically significant (specificity: 99.02%; P<0.001). Apple devices had an average specificity of 99.61% (95% CI: 98.9% to 100.32%), KB with 81.13% (95% CI: 75.19% to 87.08%), and Samsung devices with 97.96% (95% CI: 96.71% to 99.22%). Overall sensitivity across all devices was 99.02% (95% CI: 98.41% to 99.63%) (Figures 4-5).

Quality of evidence

Based on the GRADE approach, the certainty assessment was found to be high for sensitivity and specificity (Table 2). None of the studies compared two devices; each study assessed the accuracy of a single wearable device, which resulted in a serious indirectness assessment. Other considerations include one study from Apple, Inc, which was not published in a peer-reviewed journal. Quality of evidence was found to be satisfactory across all studies [18,19] (Table 3).

**Table 2:** Grading of Recommendations, Assessment, Development, and Evaluation (GRADE) Assessment of Quality of Evidence CI: confidence interval

Certainty Assessment							Number of Instances				Relative Effect (95% CI)	Certainty
Number of Studies	Study Design	Risk of Bias	Inconsistency	Indirectness	Imprecision	Other Considerations	Apple	Apple + KardiaBand		Samsung		
Sensitivity												
9	Observational Studies	Not serious	Not serious	Serious	Not serious	Not serious	814	368	1064		Apple: 97.23 (95.43, 99.03) versus Apple + KB: 96.94 (94.71-99.16) versus Samsung: 95.47 (93.10, 97.84)	◯◯◯⨁ HIGH
Specificity												
9	Observational Studies	Not serious	Not serious	Serious	Not serious	Not serious	814	368	1064		Apple: 92.98 (91.95, 94.02) versus Apple + KB: 68.09 (65.20-70.98) versus Samsung: 93.10 (92.34, 93.87)	◯◯◯⨁ HIGH

**Table 3:** Newcastle-Ottawa Scale (NOS) for Assessing Quality of Included Studies

Author Year	Representativeness of the Cohort	Ascertainment of Exposure	Outcome of Interest	Comparability of Cohorts	Assessment of Outcome	Adequate Follow-up Duration	Adequacy of Follow-up of Cohorts
Apple							
Seshadri et al. 2020 [20]	*	*	*	N/A	*	*	
Apple, Inc 2018 [21]	*	*	*	N/A	*	*	
Tison et al. 2018 [22]	*	*	*	N/A	*	*	
Apple + KardiaBand							
Wasserlauf et al. 2019 [23]	*	*	*	N/A	*	*	
Bumgarner et al. 2018 [24]	*	*	*	N/A	*	*	
Rajakariar et al. 2020 [25]	*	*	*	N/A	*	*	
Samsung							
Dorr et al. 2018 [26 ]	*	*	*	N/A	*	*	
Ding et al. 2019 [27]	*	*	*	N/A	*	*	
Bashar et al. 2019 [28]	*	*	*	N/A	*	*	

Discussion

Summary of Results

This meta-analysis compared the sensitivity and specificity of three wrist-worn wearable devices, Apple Watch, KardiaBand accessory, and Samsung, in their ability to detect AF. The main finding of this study is that wrist-worn wearable technology offers a sensitive method to detect AF, compared to standard of care telemetry (overall sensitivity: 96.99; CI: 95.77 to 98.20). Our results also indicate that the sensitivity was sustained across all three devices (Apple Watch sensitivity: 97.92, CI: 96.09 to 99.74; KB sensitivity 96.94, CI 94.71 to 99.16; Samsung sensitivity 95.47, CI: 93.10 to 97.840).

However, this research demonstrates that specificity differs significantly between device groups (overall specificity: 99.02%; P<0.001). A specificity funnel plot revealed that there might be some publication bias with regards to the KardiaBand studies. Furthermore, a specificity forest plot revealed a wide confidence interval for one of the included Apple studies. This forest plot also demonstrated that the mean of both KardiaBand studies fell short of the overall average (Figure 4).

Clinical significance and future directions

This study demonstrates clinical significance with regards to specificity between device groups, indicating that there is some discrepancy between how these device groups interpret “normal sinus rhythm” (NSR) or “not normal sinus rhythm.” This type of diagnostic information can add value as a screening tool for patients who are either at risk for AF or patients who have had a stroke and are seeking to understand whether it may have been of cardiac origin.

The majority of the studies included patients with some form of cardiac medical history, such as AF. It is important to test these devices in patients who were never diagnosed with AF. This will provide more accurate information on the potential for these devices to be used as a diagnostic screening tool.

Limitations

There are limitations that should be considered with this study. First, an indirect comparison was performed for this meta-analysis. Second, given the sample sizes, a small number of true negatives and false positives may have influenced the specificity. With this in mind, a number of false positives may have artificially inflated the specificity. Lastly, one of the studies included in this analysis was from Apple, Inc but it was not published in a peer-reviewed journal.

## Conclusions

In conclusion, this research demonstrates that wrist-worn wearable devices offer promising results in detecting AF in patients with paroxysmal AF. However, caution is needed in all three devices regarding the use of this technology to detect NSR in patients with and without a history of AF. This research suggests that more rigorous prospective data is needed to understand the limitations of these devices in regard to varying specificities which may lead to unintended downstream medical testing and costs.
